# The amygdaloid body of the family Delphinidae: a morphological study of its central nucleus through calbindin-D28k

**DOI:** 10.3389/fnana.2024.1382036

**Published:** 2024-05-30

**Authors:** Simona Sacchini, Cristiano Bombardi, Manuel Arbelo, Pedro Herráez

**Affiliations:** ^1^Veterinary Histology and Pathology, Institute of Animal Health and Food Safety (IUSA), Atlantic Center for Cetacean Research, Marine Mammals Health WOAH col Centre, University of Las Palmas de Gran Canaria, Veterinary School, Las Palmas, Spain; ^2^Department of Morphology, Campus Universitario de San Cristobal, University of Las Palmas de Gran Canaria, Las Palmas de Gran Canaria, Spain; ^3^Department of Veterinary Medical Sciences, University of Bologna, Bologna, Italy

**Keywords:** amygdaloid body, central nucleus of the amygdala, toothed whales, dolphins, calbindin-D28k

## Abstract

**Introduction:**

The amygdala is a noticeable bilateral structure in the medial temporal lobe and it is composed of at least 13 different nuclei and cortical areas, subdivided into the deep nuclei, the superficial nuclei, and the remaining nuclei which contain the central nucleus (CeA). CeA mediates the behavioral and physiological responses associated with fear and anxiety through pituitary-adrenal responses by modulating the liberation of the hypothalamic Corticotropin Releasing Factor/Hormone.

**Methods:**

Five dolphins of three different species, belonging to the family Delphinidae (three striped dolphins, one common dolphin, and one Atlantic spotted dolphin), were used for this study. For a precise overview of the CeA’s structure, thionine staining and the immunoperoxidase method using calbindin D-28k were employed.

**Results:**

CeA extended mainly dorsal to the lateral nucleus and ventral to the striatum. It was medial to the internal capsule and lateral to the optic tract and the medial nucleus of the amygdala.

**Discussion:**

The dolphin amygdaloid complex resembles that of primates, including the subdivision, volume, and location of the CeA.

## 1 Introduction

The amygdala or amygdaloid body or complex (from now on, AMY) is an heterogeneous group of at least 13 nuclei and cortical areas, some of them with two or more subdivisions, segmented into three main nuclear masses: the cortico-medial nuclear group (also called “superficial nuclei”), which originates from the parahippocampal cortex, the baso-lateral nuclear group (also known as “deep nuclei”), and the remaining nuclei or areas (Bombardi, 2014). The deep, superficial, and the remaining nuclei have various cytoarchitectonic, chemoarchitectonic, and connectional properties. The AMY is a highly ordered region that can convert sensory data into emotions and play a key role in fear and anxiety (LeDoux, 2003). It acts on the hypothalamus and the brain systems that regulate behavior, causing the freezing, alertness, and fear responses that precede the “fight or flight” response. The structure and size of the AMY is directly associated with the complexity of structures and social relationships, according to studies carried out in humans (Bickart et al., 2011).

The central nucleus of the amygdala (CeA) is located dorso-medially to the rostral part of the AMY, bordered laterally by the basolateral complex, dorsally by the globus pallidus and medially by the stria terminalis. The CeA has four divisions in the rat (capsular, lateral, intermediate, and medial) and two in humans and primates (lateral and medial) (McDonald, 1982; Jolkkonen and Pitkanen, 1998; Bombardi, 2014). Morphologically, its neurons are reminiscent to those of the striatum. Similar to this, the projections from the CeA are GABAergic, while the glutamatergic projections are located in the basolateral region, as is the case with most striatal neurons (Davis et al., 1994). CeA plays a fundamental role in generating negative emotional responses (fear and anxiety) to environmental stimuli (Davis, 1997) and is well connected with the autonomic and endocrine centers of the brainstem and hypothalamus (Sorvari et al., 1996).

The basolateral AMY receives sensory input from the thalamus and cortex (McDonald, 1998) and sends abundant glutamatergic projections to the CeA and the Bed Nucleus of the Stria Terminalis (BNST) (Pitkanen et al., 1995), being fundamentally related to fear conditioning (Phelps and LeDoux, 2005) and its extinction (Quirk and Mueller, 2008). The Corticotropin Releasing Factor/Hormone, a 41-amino acid peptide, plays a fundamental role in hormonal, behavioral, and sympathetic responses to stress (Sakanka et al., 1986). Its receptors are abundant in the CeA, in the Bed Nucleus of the Stria Terminalis and in the basolateral AMY (Lee et al., 2008). The CeA and the Corticotropin Releasing Factor seem to play a fundamental role in the translation of negative mood states into anxiety-related attitudes, and even promote alcohol dependence (Gilpin, 2012).

The two taxonomic suborders of cetaceans are the toothed whales (Odontoceti) and the baleen whales (Mysticeti). Mammals formerly classified as artiodactyls and cetaceans have been recently combined into the clade *Cetartiodactyla* due to their close phylogenetic relationship. The extraordinarily large brains, both in absolute and relative terms, and the incredibly dense folding of the neocortex are two distinctive features of toothed whales. The AMY of the toothed whales seems highly developed as in other mammals (Oelschläger and Oelschläger, 2002; Marino et al., 2004b). It consists of an olfactory (paleocortical) component and a basal ganglia component. Nonetheless, prior studies on cetacean AMY are scarce and most of them are from manuscripts written in the latter half of the previous century. Breathnach and Goldby (1954) described the AMY in the porpoise (*Phocoena phocoena*; Linnaeus, 1758; family Phocoenidae), while Jansen (1953) provided information of the fin whale (*Balaenoptera physalus*; Linnaeus, 1758; family Balaenopteridae). Morgane and Jacobs (1972) published a relatively more recent contribution, which presented a macroscopical description in the bottlenose dolphin (*Tursiops truncatus*, Montagu 1821, family Delphinidae), taking as reference the two contributions by Breathnach and Jansen. The greatest limitation in referencing these works, is that the nomenclature on the parcellation of the AMY has been evolving in the last decades, differing from the previous classification. In fact, earlier classifications were based on Johnston’s (1923) proposal, where for the first time an “amygdaloid complex” is named, composed of only six major nuclei (basal, basal accessory, lateral, central, medial, and cortical), as well by the intercalated cell masses and the nucleus of the lateral olfactory tract (NLOT). The previous publications on cetaceans’ AMY (Jansen, 1953; Breathnach and Goldby, 1954; Morgane and Jacobs, 1972) were based on the following classification made in 1941 (Crosby and Humphrey, 1941):

• the basolateral amygdaloid nuclei, constituted by the lateral, basal, basal accessory, and intercalated nuclei;
• the cortico-medial amygdaloid nuclei represented by the cortical, medial, and central nuclei, the terminal stria and, according to Breathnach and Goldby (1954), also by the anterior amygdaloid area.

The aforementioned classification is very different from the current parcellation of the AMY (Pitkänen and Kemppainen, 2002; Bombardi, 2014), which is based on the names that Price first proposed in 1987 (Price et al., 1987).

In an effort to characterize the structure’s overall morphology, focusing on the CeA, the present study analyzed the entire AMY of five dolphins of three different species.

## 2 Materials and methods

This investigation relies solely on post-mortem analysis, so ethical review and approval were not required. The Spanish Ministry of Environment and the Canary Islands Government’s environmental department granted the necessary authorization for the management of stranded cetaceans. Live animals were not used in any of the experiments.

### 2.1 Tissue preparation

Brains were obtained from five specimens of three different species of the suborder Odontoceti, belonging to the family Delphinidae and stranded in the Canary Islands between 2005 and 2011 (Sacchini, 2015): three striped dolphins (*Stenella coeruleoalba*, Meyen 1833), one common dolphin (*Delphinus delphis*, Linnaeus 1758), and one Atlantic spotted dolphin (*Stenella frontalis*, Cuvier 1829). While cross-sections were used to study the histology of the AMY, cross, sagittal, and horizontal sections of whole brains were also made to provide a more accurate assessment of the AMY’s topography within the brain. For this reason, three Atlantic spotted dolphins and one Blainville’s beaked whale (*Mesoplodon densirostris*, De Blainville 1817, family Ziphiidae) were added to the research, and their brains were examined using sagittal and horizontal sections solely for macroscopical purposes, revealing the corresponding AMY. The AMY macroscopical localization and relationship to surrounding structures were all revealed by each cutting plane. A comprehensive protocol for sample collection, histochemical and immunohistochemical procedures, and brain handling is available from a previous published work (Sacchini et al., 2022). Briefly, at the time of necropsy, brains were extracted and coded for freshness (Decomposition Code ≤2, fresh). Both the cerebral and cerebellar hemispheres underwent some longitudinal incisions prior to immersion. Most of the cuts were superficial, but at least one of them went deep into each cerebral hemisphere to reveal the lateral ventricles and let the fixative enter the ventricular system. Brains were submerged in 4% formaldehyde in phosphate-buffered saline (PBS; pH 7.4), during at least 72 h. Subsequently, one or both entire AMY from each animal (Table 1) were isolated, postfixed during 24 h, and then cryoprotected by immersion in a 30% sucrose and 0.1% sodium azide solution in PBS (pH 7.4), at +4°C, to prevent freezing artifacts. Samples were cut rostrocaudally, using a freezing sliding microtome (Leica SM 2000 R, University of Bologna). Sections were stored in PBS containing sodium azide (0.01%). The high quality of the tissues was confirmed by thionine staining. Every fourth serial section was collected for Nissl staining (thionine) and the adjacent sections for calbindin-D28k (c-D28k) free-floating immunohistochemistry. Of all the sections obtained, 13 were chosen as main references and as equidistant as possible, in order to obtain common references to each AMY.

**TABLE 1:** A summary table of the dolphins utilized in this research.

Species	Found	Sex	Age	Length (cm)	Sampled amygdala
*Stenella coeruleoalba*	Alive	F	Y	168	Right
*Stenella coeruleoalba*	Alive	F	A	205.5	Right
*Stenella coeruleoalba*	Alive	M	A	209	Both
*Delphinus delphis*	Dead	M	A	227	Right
*Stenella frontalis*	Dead	M	A	182	Both

F, female; M, male; A, adult; Y, young.

### 2.2 Immunohistochemistry

Immunoperoxidase staining procedure was carried out on free-floating 50-μm–thick coronal sections under moderate shaking conditions. Formalin fixed free-floating sections were treated with 3% H_2_O_2_ in PBS for 30 min at room temperature (RT), to quench the endogenous peroxidase activity, and rinsed in PBS (three times, 10 min each). To block non-specific binding, sections were incubated in a solution containing 10% normal horse serum (Vector, S-2000), containing 0.5% Triton X-100 (Merck, Darmstadt) to permeabilize the tissue, in PBS for 2 h at RT. Thereafter, a first set of sections was incubated in a mouse monoclonal anti c-D28k (Swant, Code N° 300), diluted 1:500, overnight at +4°C. The following day, the sections were rinsed in PBS (three times, 10 min each) and incubated for 45 min at RT with a secondary antibody biotinylated horse anti-mouse (Vector Laboratories, BA-2000, diluted 1:200) in a solution containing 1% normal horse serum in PBS. The sections were rinsed in PBS and incubated with an avidin-biotin complex, (ABC, Vector Laboratories; PK-4000) for 1 h at RT. Finally, peroxidase activity was revealed with a 3,3′-diaminobenzidine peroxidase kit (Vector Laboratories, SK-4100). The sections were mounted onto gelatine-coated slides and dried overnight at RT. Slides were then dehydrated in ethanol, cleared in xylene and coverslipped with Entellan (Merck, Darmstadt, Germany).

### 2.3 Specificity of the calbindin-D28k

Calbindin-D28k is a calcium-binding protein of the EF-hand (helix-loop-helix motif) family related to calmodulin and troponin-C. Through their roles as Ca^2+^ transporters across cell membranes, Ca^2+^-modulated sensors, or decoding Ca^2+^ signals, calcium-binding proteins, and thus c-D28k, contribute to the regulation of Ca^2+^ concentration in the cytosol and take part in a variety of biological functions (Yáñez et al., 2012). Monoclonal c-D28k is a mouse IgG1 generated through hybridization between mouse myeloma cells and mice’s spleen cells that were immunized with purified c-D28k from chicken. The amino acid sequence of the c-D28k was compared with the mouse. The Ensembl genome browser^1^ shares comparative genomics and sequence variation. The sequence of c-D28k is shared with the mouse (*Mus musculus*) for over 96% in the following cetaceans: bottlenose dolphin, beluga whale (*Delphinapterus leucas*; Pallas 1776; family Monodontidae), sperm whale (*Physeter macrocephalus*; Linnaeus 1758; family Physeteridae), and even in the blue whale (*Balaenoptera musculus*; Linnaeus, 1758; family Balaenopteridae). Specifically, the protein alignment between the mouse and the bottlenose dolphin is: 261 amino acids length, 96% protein identity, and 100% coverage. To evaluate the specificity of the secondary antibody, negative controls were conducted using only PBS and the secondary antibody instead of the primary antibody.

### 2.4 Neuronal morphometric study

In order to conduct the neuronal morphometric study, neurons were photographed at 40× magnification, using the Olympus XC50 digital camera. The measures were conducted using a digital image software (CellSens Standard, Olympus). In the thionine-stained sections, neurons that exhibited a distinct border, without any blurring, and a discernible nucleolus were identified for measurement. Neural populations were classified using indicators such as dendritic count, neuron size and shape and grouped into four groups: polygonal, spherical, fusiform, and large polygonal. A grade of affinity to thionine (weak, moderate, or strong) was also assigned to each cell, and the perikaryal area and perimeter were measured. The data were presented as mean ± standard deviation (SD).

## 3 Results

### 3.1 Macroscopical organization of the amygdaloid body

**FIGURE 1:**
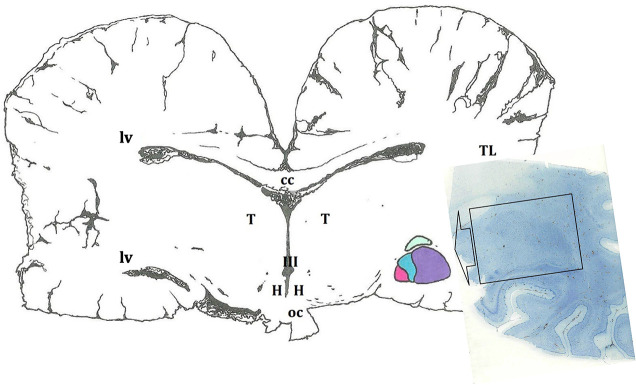
Schematic representation of the amygdaloid complex (in colors), in a cross section of the brain obtained at the level of the hypothalamus (H). In this diagram, as in the rest of the cross sections, a certain asymmetry of the two hemispheres predominates. The microscopic image depicts a histological preparation of the amygdala (enclosed by a black box). III, third ventricle; cc, corpus callosum; oc, optic chiasm; T, thalamus; TL, temporal lobe; lv, lateral ventricle.

**FIGURE 2:**
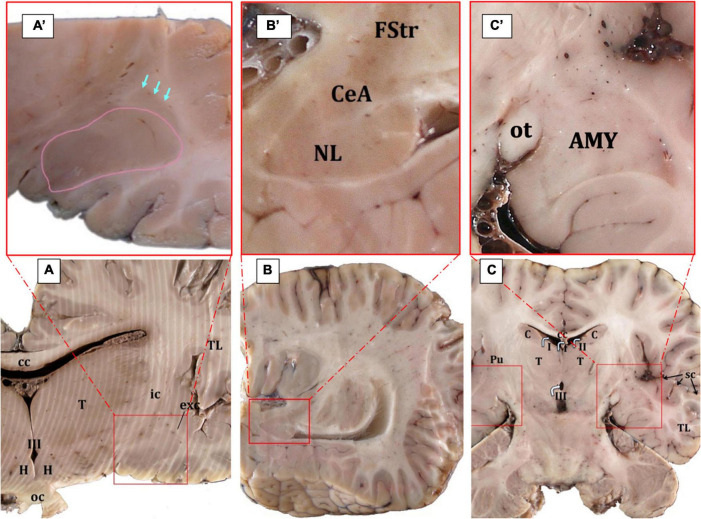
Gross anatomy of the AMY (surrounded by red squares) and the adjoining structures **(A–C)**. The edges of the central nucleus are indicated by arrows while the basolateral AMY in surrounded by a pink line; Cuvier’s beaked whale, transverse plane **(A,A’)**. The central nucleus is located ventral to the *fundus striati* and dorsal to the lateral nucleus; Atlantic spotted dolphin, parasagittal plane **(B,B’)**. In the horizontal plane, the AMY is also clearly evident; Atlantic spotted dolphin **(C,C’)**. AMY, amygdaloid body; cc, corpus callosum; C, caudate nucleus; CeA, central nucleus of the amygdala; exc, extreme capsule; FStr, fundus striati; H, hypothalamus; ic, internal capsule; oc, optic chiasm; ot, optic tract; Pu, putamen; sc, sylvian cleft; T, thalamus; TL, temporal lobe; I, II, III, first, second, and third ventricle.

By identifying neighboring structures, all types of sections—transverse, sagittal, and horizontal—provided information on the macroscopic morphology of the AMY and made it possible to establish the rostro-caudal, dorso-ventral, and medio-lateral limits. The AMY appeared as a well-defined, almond-shaped structure of a considerable relative size if compared to the total brain volume (Figures 1, 2). Its approximate size was measured only in the Atlantic spotted dolphin and had a mean of 1.3 cm (length) for 2 cm (wide).

In serial cross sections of the brain, just at the level of the hypothalamus (Figure 2A), the AMY was lateral to the diencephalon (thalamus and hypothalamus), separated from them by a thick internal capsule. Medially and ventrally, the optic tract was evident. The neocortex of the temporal lobe and the sylvian fissure were observed laterally to the AMY. A very thin external capsule separated the AMY from a thin, sheet-like neuronal structure, the claustrum, observed only rostrally and just at the beginning of the AMY. On the other side, a thin extreme capsule separated the claustrum and the AMY from the neocortex of the temporal lobe (Figures 2A, A’). Dorsally, the *fundus striati* (FStr), the most ventral part of the striatum, was present and this was quite evident through parasagittal sections (Figures 2B, B’). The AMY extended from the caudal limit of the claustrum to the rostral limit of the hippocampal formation and the lateral ventricle. Therefore, the hippocampal formation and the onset of the lateral ventricle were observed, marking the caudal limit of the AMY (Figures 2B, B’, C, C’). Figure 2B’ will be used to suggestively sequence the development of the CeA.

### 3.2 Microscopical organization of the amygdaloid body

Microscopically, the AMY appeared as a heterogeneous grouping of brain nuclei, some of which were located rostrally, while others caudally. Twelve nuclei divided into three main groups were observed. The basolateral complex (deep nuclei or basolateral amygdala) was the largest and composed by:

• Lateral nucleus (NL);
• Basal nucleus (NB) with its magnocellular, intermediate, and parvicellular divisions;
• Basal accessory nucleus (NBA) with its magnocellular and parvocellular divisions;
• Paralaminar nucleus (PL).

Pyramidal and non-pyramidal neurons in all deep nuclei presented a disorganized distribution.

The cortical or superficial area were made up of:

• Anterior cortical nucleus (CoA);
• Posterior cortical nucleus (CoP);
• Periamygdaloid area/cortex (PAC);
• Medial nucleus (M).

With the exception of the medial nucleus, the pyramidal and non-pyramidal neurons of the superficial nuclei presented a tri-stratified arrangement.

The remaining areas were composed by:

• the anterior amygdaloid area (AAA);
• the amygdalo-hippocampal area (AHA);
• Intercalated nuclei or cell masses (I);
• the central nucleus of the amygdala (CeA).

The neurons of the remaining areas were similar to those of the striatum or FStr, without organization in layers, except for the AHA in which a stratified distribution was observed, with the presence of pyramidal and non-pyramidal neurons.

In the examined dolphins, neither the nucleus of the lateral olfactory tract (NLOT) nor the bed nucleus of the accessory olfactory tract (BAOT) were identified.

### 3.3 Microscopical arrangement and boundaries of the amygdaloid body

#### 3.3.1 The basolateral complex

**FIGURE 3:**
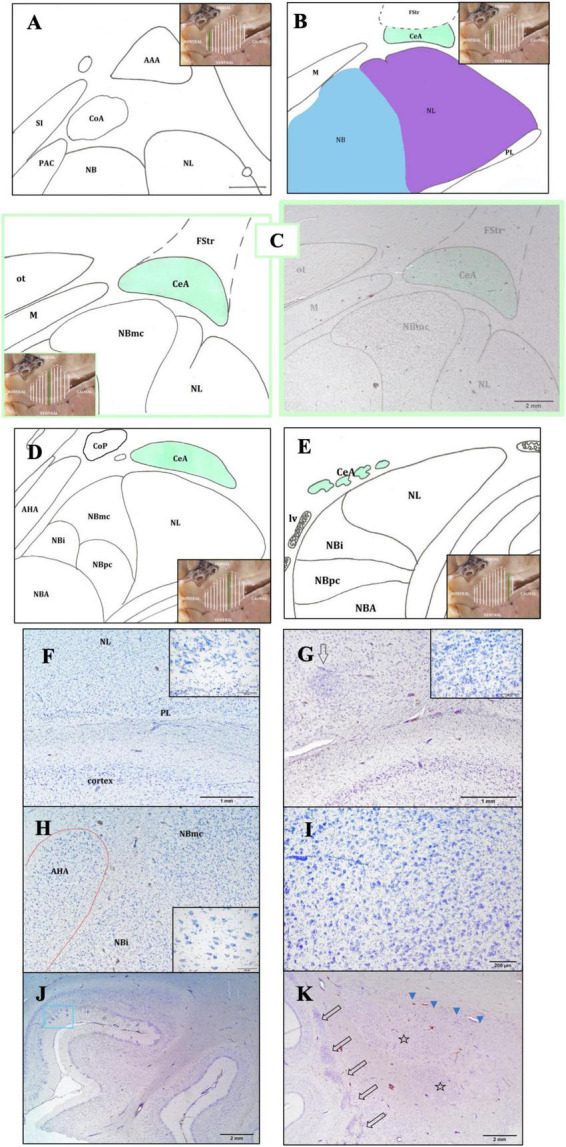
Rostro-caudal description of the CeA through schematic representations. The purpose of the insets is to aid the reader in comprehending the precise position and evolution of the AMY and CeA **(A–E**). In the image, the following structures are observed: the lateral nucleus (NL), the basal nucleus (NB); the periamygdaloid cortex (PAC); the anterior cortical area (CoA), dorsal to the PAC; the anterior amygdaloid area (AAA), dorsal to the abovementioned nuclei; the *substantia innominata* (SI), medial to the amygdaloid complex **(A)**. Caudally, the CeA is relatively elongated in shape, dorsal to the NL (in purple) and ventral to the *fundus striati* (FStr); the medial nucleus (M) is medial to the CeA; the NL is ventral to the CeA and lateral to the NB (in blue). The NB and the paralaminar nucleus (PL) are also represented **(B)**. Maximum size of the CeA. The magnocellular division of the NB (NBmc) has a more rounded shape, reflected by its great extension and its large neurons. The optic tract (ot) which is situated medial to the CeA, dorsal to the M, and dorsal-medial to the NBmc, is clearly visible **(C)**. Caudally, both the CeA and the NBmc are diminished. The whole NB has a significant reduction in all three of its subdivisions, included the intermediate (NBi) and parvicellular (NBpc) divisions, but NBmc is the most affected. The NL shows the disappearance of its small accessory portion. Two additional superficial nuclei are also present: the posterior cortical nucleus (CoP) and the anterior amygdalohippocampal area (AHA) **(D)**. Finally, only isolated islets of CeA neurons are visible at this stage, and its perimeter and area cannot be ascertained. Only the deep nuclei—the NB, NL, and NBA—and the beginning of the lateral ventricle (lv) are visible, aside from the CeA **(E)**. Bright-field photomicrograph of Nissl-stained sections of the lower part of the AMY **(F,G)**. Histological images of the PL with its neuronal morphology (inset) **(F)** and the intercalated cell masses (arrow) and their neuronal morphology (inset) **(G)**; common dolphin, rostral right AMY, thionine. Histological image of the anterior amygdalohippocampal area (AHA), located medially to the NB, and its large neurons, striped dolphin, caudal right AMY, thionine **(H)**. The histological image displays the magnocellular division of the NB (NBmc), rostral right AMY, striped dolphin, thionine **(I)**. Entorhinal cortex histology, ventral to the AMY. Neurons with polygonal cell bodies and dark staining are seen in layer II; these neurons are typically grouped into islands (blue box), common dolphin, thionine **(J)**. Histological images of the caudal left claustrum, whose segmented appearance (arrows) and non-stratified neuronal arrangement define its caudalmost portion. The NL mostly represents the first part of the AMY (stars) and is accompanied by the *substantia innominata* (arrowheads); Atlantic spotted dolphin, thionine **(K)**.

**FIGURE 4:**
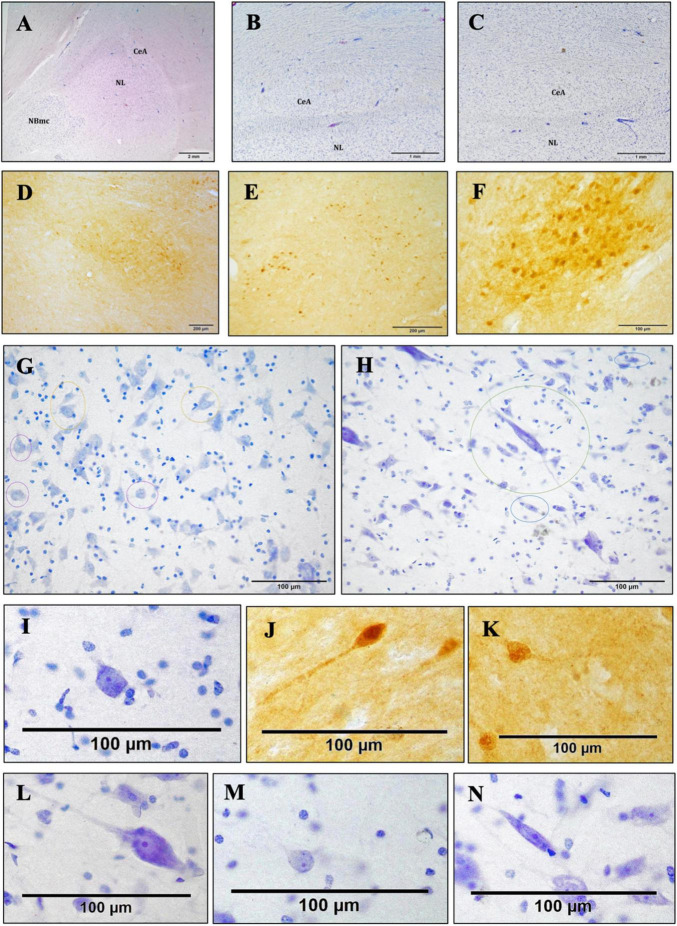
Bright-field photomicrograph of Nissl-stained and c-D28k immunoreactive sections showing the cytoarchitectonics of the CeA. Histological image of the right AMY and CeA of an Atlantic spotted dolphin. Comparing this species to the others, the CeA showed a lower dimension, despite having reached its highest size. In fact, the CeA does not extend to the NBmc; rather, it is solely dorsal to the NL, thionine **(A)**. Histological image of the CeA, which is characterized by the presence of nerve fiber bundles and a very reduced size. Common dolphin, caudal right AMY, thionine **(B)**; striped dolphin, caudal right AMY, thionine **(C)**. The CeA exhibits immunoreactivity against c-D28k. Atlantic spotted dolphin, rostral right AMY **(D)**; striped dolphin, caudal left AMY **(E)**; common dolphin, rostral right AMY **(F)**. Neurons of different morphologies; thionine **(G,H)**. Poligonal (yellow circles), round (purple circles) Atlantic spotted dolphin **(G)**. Fusiform (blue circles), LP (green circle), striped dolphin **(H)**. Poligonal neurons, thionine **(I)** and c-D28k free-floating immunohistochemistry **(J,K)**. LP **(L)**. Round **(M)**. Fusiform **(N)**.

The NL was characterized by a densely packed structure, consisting of large neurons, which showed intense staining to thionine. It was lateral to the NB and medial to the external capsule. In general, it consisted of medium-sized and medium-large cells. It was the first to appear and was present throughout the entire AMY extension (Figures 3A–E, G, K). It was possible to distinguish a principal and a smaller accessory division that were positioned medially to the main portion (not shown). Situated medially to the NL, the NB presented three divisions: parvicelular (small neurons), intermediate (medium size neurons), and magnocellular (large neurons) (Figures 3D, E, H, I). Because the neurons in the magnocellular division (from now on, NBmc) were large and had a high thionine staining intensity, the division was easily distinguishable (Figures 3H, I, 4A). Compared to the other amygdaloid nuclei, the NBmc and the NL were identified by the size of their neurons and their strong thionine staining affinity. The NBA presented two subdivisions, parvicellular and magnocellular, and was located between the NB and the cortical nuclear group (Figures 3D, E). The PL was located ventral to the NL, on the ventral side of the basolateral nuclear group (Figure 3B). It was flattened and elongated and its neurons were disorganized (Figure 3F). The entorhinal cortex was found ventral to the PL (Figure 3J).

#### 3.3.2 The cortical or superficial area

The CoA was located in the rostral portion of the AMY, dorsally to the PAC and medially to the basolateral complex. The CoP was located in the caudal portion of the AMY, dorsal to the AHA (Figure 3D). The PAC was situated in the rostral half of the AMY, medially, in between the substantia innominata and the basolateral complex (Figure 3A). Pyramidal and non-pyramidal medium-size neurons with a moderate affinity to the thionine were observed in the PAC, grouped in layers with a vertical and parallel arrangement. The M was medial to the basolateral complex (Figures 3B, C) and was characterized by small neurons, arranged parallelly.

#### 3.3.3 The remaining areas

The AAA was seen in the first part of the AMY, before the formation of the CeA, was dorsal to most of the aforementioned nuclei (Figure 3A) and distinguished by its disorganized neurons resembling striatal neurons. The I were identified as clusters of small neurons dispersed among the other nuclei, primarily dorsally to the NL and between the NB and NL (Figure 3G). The enlarged form and parallel distribution of its neurons made the AHA, situated medial and parallel to the NB and easy to identify (Figures 3D, H). Section “3.4 The central nucleus of the amygdala” provides a thorough description of the CeA.

The beginning of the AMY, specifically the NL, corresponded to the most caudal limit of the claustrum (Figure 3K, arrows), which is characteristic for its segmented appearance and non-stratified arrangement of its neurons. Similarly, at this point, the following structures were seen in a medio-dorsal position to the NL: the substantia innominata, medio-dorsally to the AMY, was distinguished by its large neurons that were oriented in cords and intensely stained with thionine (Figure 3K, arrowheads); the striatum, dorsally, formed by the putamen and the caudate nucleus; and the internal capsule, which was made up of bundles of white matter. Microscopically, the entorhinal cortex was visible ventrally to the AMY due to its characteristic narrow layer II, which was formed by closely spaced neurons that formed islets (Figure 3J, blue box). Dorsally, the FStr, the most ventral part of the striatum, was present (Figures 2B’, 3B, C).

### 3.4 The central nucleus of the amygdala

#### 3.4.1 Morphology and boundaries

The schematic representations in Figures 3A–E are meant to depict the rostrocaudal evolution of the CeA. Along with the M, the CeA of the dolphins under examination was seen caudally to the AAA (Figure 3A). CeA extended mainly dorsal to the basolateral complex and ventral to the FStr (Figures 3B, C). It was located lateral to the internal capsule, the optic tract, and the M. The rostral limit of the CeA coincided then with the caudal limit of the AAA (Figures 3A, B). At this stage, the CeA was visible as a small cluster of neurons positioned dorsal to the NL and ventral to the caudalmost part of the AAA (not shown). The onset of the CeA was noticed, along with the development of the NBmc. In fact, the CeA was larger and took on a more pronounced structure caudalward, in accordance with the NBmc increasing size. In the common dolphin, the CeA’s approximate area at this level was 102,475,8.3 μm^2^ and its approximate perimeter was 14,509.8 μm (Table 2). Because of its close continuity to the FStr, situated dorsally to the CeA, it was challenging to identify the CeA’s dorsal boundaries at this stage of the AMY development. Despite that and in contrast to the CeA, where the arrangement of the neurons was random, the striatal neurons had a more ordered distribution. However, the segmented appearance of the FStr in the species Atlantic spotted dolphin made it easier to distinguish between the CeA and FStr. The CeA was situated laterally to the M and ventro-medially to the FStr, just like the other species, but it had more distinct boundaries (Figure 4A). The CeA expanded dorsally and medially, eventually reaching the NBmc. Simultaneously, a strong continuity with the M was noted, distinctly defining the boundaries between the two based on their respective neuronal distributions: random in the CeA and parallel in the M. The CeA reached its maximum extension at the same time as the NBmc (Figure 3C), reaching in the common dolphin an area of 1,753,491.8 μm^2^ and a perimeter of 19,771 μm (Table 2). Bundles of white matter began to subdivide the CeA, which, in this way, began to be organized into well-defined groups of neurons (Figures 4A–C). Nonetheless, this was the point at which the CeA of the species Atlantic spotted dolphin differed from other species. Here, the CeA reached its maximum volume; however, due to its dorsal confinement to the NL, it never grew to the size of other species. Unlike other species, it never reached the NBmc (Figure 4A). Caudally, the size of the AMY decreased considerably, flattening dorsoventrally (Figure 3E). The deep nuclei (lateral, basal, basal accessory, and paralaminar) were also considerably reduced. Bundles of white matter separated the CeA from the NL (Figures 4B, C). Hence, caudally advancement resulted in an increase in white matter bundles, which divided the CeA into consistently smaller and fewer neuronal groups. The very small CeA that remained vanished along with the NBmc (Figures 3D, E) and the formation of the CoP and the AHA (Figure 3D) and finally the lateral ventricle (Figure 3E). c-D28k immunochemistry provided proof of the rostro-caudal development of the CeA (Figures 4D–F).

**TABLE 2:** Areas and perimeters of the CeA of the common dolphin, along its rostro-caudal development.

Position inside the AMY 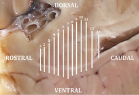	Area (μm^2^)	Perimeter (μm)
1	No CeA	
2		
3	1,024,758.3	14509,8
4	1,140,161.5	14,660.9
5	1,330,824.2	15,332.4
6	1,537,547.3	19,687.6
7 (maximum size of the CeA)	1,753,491.8	19,771
8	1,258,136.4	19,045.3
9	98,504.7	15,986.9
10	413,751.1	13,319.6
11	293,281.5	9,694.2
12	Only small fragments of CeA	
13	No CeA	

Figure 2A’ is used for the sequencing of the AMY. The limits of the CeA were frequently poorly defined, so any estimate of the its size is only approximate.

#### 3.4.2 Cytoarchitecture

Neurons with a range of morphologies, including in the following order polygonal < round < fusiform, were unevenly distributed in the CeA (Figures 4G, H). These were tiny neurons with a weak thionine staining. Additionally, scattered, highly stained, large polygonal neurons (LP) were randomly arranged in the CeA (Figure 4H, green circle) and in the band of white matter confined between the CeA and the NL. In the caudal half of the CeA, LP neurons were diminishing. Two striped dolphins and one common dolphin, were selected for the morphometry of the neuronal types to ascertain whether there were significant inter-specific variations, confirming that cell sizes were similar rather than variable among the two Delphinidae.

##### 3.4.2.1 Polygonal neurons

The body, or soma, of the polygonal neurons was asymmetrically shaped, either triangular or quadrangular (Figures 4G, I–K). Quadrangular neurons had one axon and three dendrites, while triangular neurons had one axon and two dendrites. The neurons displayed a centrally located nucleus, a clearly visible nucleolus, and uniformly distributed Nissl or tigroid bodies. For this particular neuronal morphotype, the perimeter ranged from 40 to 100 μm, and the mean neuronal area varied between 100 and 450 μm^2^. The SD and average were 218.52 ± 63.73 μm^2^ for the area, and 63.81 ± 10.35 μm for the perimeter. In addition, LP neurons were observed to be significantly larger (Figure 4L). The LP neurons were characterized not only by their size but also by their strong thionine affinity. These neurons were distinguished by occasionally having three or more oligodendrocytes surrounding them. The LP average neuronal area was found to be highly variable, measuring between 450 and 1,800 μm^2^ with a perimeter between 70 and 200 μm. The area’s average and SD were 770.59 ± 320.04 μm^2^, while the perimeter’s was 124.96 ± 30.34 μm.

##### 3.4.2.2 Round neurons

Round neurons had a spherical soma with two dendrites and an axon as well as three dendrites, though thionine staining did not always show all three processes (Figures 4G, M). This neuronal group additionally exhibited a central nucleus, a clearly visible nucleolus, and uniformly distributed Nissl bodies within the perikaryon. For this particular neuronal morphotype, the perimeter ranged from 40 to 80 μm, while the mean neuronal area varied between 100 and 300 μm^2^. The area’s average and SD were 163.68 ± 44.06 μm^2^, and the perimeter’s was 49.63 ± 6.32 μm.

##### 3.4.2.3 Fusiform neurons

The soma of the fusiform neurons was long and spindle-shaped (Figures 4H, N). They frequently showed two dendrites, one at each extremity of the neuronal soma, a centrally located nucleus, a clearly visible nucleolus, and uniformly distributed Nissl bodies throughout the perikaryon. For this particular neuronal morphotype, the perimeter ranged from 40 to 80 μm, while the mean neuronal area varied between 100 and 300 μm^2^. The area’s average and SD were 174.56 ± 39.62 μm^2^, while the perimeter’s values were 65.06 ± 10.73 μm.

#### 3.4.3 Calbindin-D28k immunoreactivity

The boundaries of the CeA were more precisely defined and identified through the immunohistochemistry against calbindin-D28k (Figures 4D–F). This region was found to have a triangular shape and exhibit high immunoreactivity in the neuropil. While positivity against this neuromarker was also expressed in regions close to the CeA, such as the striatum, the substantia innominata, and other amygdaloid nuclei like the intercalated cell masses, the intensity of the CeA immunoreactivity was different from that of other nearby areas. However, on occasions, the immunolabeling was quite heterogeneous and was not expressed throughout the entire CeA, marking the soma of some neuronal groups, mainly polygonal neurons. Furthermore, neuronal immunoreactivity was not expressed homogeneously in the three neuronal morphotypes, but preferentially in polygonal neurons and, to a lesser extent, in LP neurons (Figures 4J, K). The soma was labeled homogeneously and, occasionally, also one or more dendrites. Sporadically, a certain labeling was also observed in some fusiform and spheroidal neurons. Nonetheless, polygonal neurons exhibited a greater expression and more uniform immunoreactivity than LP. In particular, the soma showed uniform markings of one or more dendrites, though not always. There was heterogeneity in the CeA immunoreactivity of the neuropil and neurons (Figures 4D–F). c-D28k-immunopositive polygonal neurons had a mean neuronal area of 100–400 μm^2^ and a mean neuronal perimeter of 40–110 μm. The area’s average and SD were 229.15 ± 61.71 μm^2^, while the perimeter’s values were 68.18 ± 14.27 μm.

## 4 Discussion

The current study examined the complete AMY of five dolphins from three distinct species in an attempt to describe the structure’s general architecture with a focus on the CeA. A brief reflection on the current nomenclature employed in the various animal species is required. The present parcellation of the AMY (Pitkänen and Kemppainen, 2002; Bombardi, 2014), which is based on the nomenclature that Price first proposed in 1987 (Price et al., 1987), is substantially different from anterior classifications. The AMY was first discovered by Burdach in the 19th century (Burdach, 1819–1826). This author described a group of cells, now known as the basolateral complex. From there, over time, other nuclei were observed that together form part of that heterogenous structure of more than 10 nuclei in the form of almond: the amygdaloid body. The amygdaloid body (lat. *corpus amygdaloideum*) is also known with the synonyms amygdaloid complex (lat. *complexus amygdaloides*) or simply amygdala (FIPAT, 2017; ICVGAN, 2017). An accurate categorization of the AMY and all neuroanatomic structures is indicative of a correct comprehension of each structure’s functionality (McDonald, 2003). This idea has revolutionized the classification of the AMY in more recent studies conducted on humans and animals. The reason for the delay in comprehending the morphology, connections, and functions of this structure is its complexity (Sah et al., 2003). In cetaceans, as in primates (Fudge and Tucker, 2009), the process of neocorticalization moves the amygdaloid complex from a vertical arrangement, as seen in other mammalian species, to an horizontal plane. In this way, nuclei such as the lateral nucleus, which in other species are located in dorsal and lateral position, in dolphins acquire a more medial and ventral situation (Jacobs et al., 1979). This may be part of the reason for which we found a different organization, distribution and therefore also nomenclature of the analyzed nuclei (Table 3).

**TABLE 3:** Nuclei of the animal and human amygdala, according to different sources.

International Neuroanatomical Terminology, FIPAT (2017) (human)	International Committee on Veterinary Gross Anatomical Nomenclature–Nomina Anatomica Veterinaria, ICVGAN (2017) (animal)	Pitkänen and Kemppainen (2002) (rat, monkey, and human)	Proposed classification for cetaceans according to Bombardi (2014)
CORPUS AMYGDALOIDEUM/AMYGDALOID BODY	CORPUS AMYGDALOIDEUM	AMYGDALOID COMPLEX	AMYGDALOID COMPLEX
Basolateral nuclear group		**Deep Nuclei**	**Deep nuclei**
**Basolateral amygdaloid nucleus**	**Nucleus basalis**	**Basal nucleus**	**Basal nucleus (NB)**
**Basomedial amygdaloid nucleus**			
Amygdaloclaustral transition area			
**Lateral amygdaloid nucleus**	**Nucleus lateralis**	**Lateral nucleus**	**Lateral nucleus (NL)**
		Accessory basal nucleus	Accessory basal nucleus (NBA)
		Paralaminar nucleus	Paralaminar nucleus (PL)
**Centromedial nuclear group**		**Superficial nuclei**	**Superficial nuclei**
		Bed nucleus of the accessory olfactory tract	*Not detected*
		Nucleus of the lateral olfactory tract (NLOT) □	*Not detected*
		Anterior amygdalohippocampal area -	
		Anterior cortical nucleus **‡**	Anterior cortical nucleus (CoA) **‡**
Central amygdaloid nucleus *	Nucleus centrales *	Posterior cortical nucleus **‡**	Posterior cortical nucleus (CoP) **‡**
**Medial amygdaloid nucleus**	**Nucleus medialis**	**Medial nucleus**	**Medial nucleus (M)**
Intercalated amygdaloid nuclei **§**			
Amygdalostriatal transition area			
		Periamygdaloid area ∙	Periamygdaloid area (PAC) ∙
**Extended amygdala**		**Remaining areas**	**Remaining nuclei**
Bed nucleus of stria terminalis (Lateral + medial divisions)			
Sublenticular extended amygdala			
Interstitial amygdaloid nucleus			
		Intercalated nuclei **§**	Intercalated nuclei (I) **§**
		Central nucleus *	Central nucleus (CeA) *
**Olfactory amygdala**			
**Anterior amygdaloid area**		**Anterior amygdaloid area**	**Anterior amygdaloid area (AAA)**
Anterior cortical nucleus **‡**	Nucleus corticalis **‡**		
Posterior cortical nucleus **‡**			
Ventral cortical nucleus			
Nucleus of lateral olfactory tract □	Nucleus tractus olfactorii lateralis □		
Amygdalohippocampal transition area -			Anterior amygdalohippocampal area (AHA) -
Amygdalo-piriform transition area			
Periamygdaloid cortex ∙			
		Lateral capsular nuclei	*Not detected*

Symbols are used to represent equivalent nuclei that are categorized differently. The nuclei that exhibit a specific coincidence across the different classifications are underlined.

### 4.1 The amygdaloid complex in the dolphin

The examined dolphins possessed a well-developed AMY that comprised the basolateral and superficial nuclear groups as well as the remaining amygdaloid nuclei, as seen in other mammals. Contrarily to Patzke et al. (2015), and in common with what described by other authors in different species of cetaceans (Jansen, 1953; Schwerdtfeger et al., 1984; Buhl and Oelschläger, 1988; Marino et al., 2003, 2004a; Marino, 2004; Tartarelli and Bisconti, 2006; Oelschläger and Oelschläger, 2009; Rambaldi et al., 2017), the AMY had a considerable size, compared to the total brain volume. The inferior horn, located in the temporal lobe of the hemisphere, is reached by the caudate nucleus, which travels along the lateral ventricle from the anterior horn. Here, the AMY and the tip of the sharp caudate nucleus can occasionally be continuous (*fundus striati*) (Cozzi et al., 2017). It is remarkable how large the AMY is in dolphins considering the overall diminished state of their olfactory system. In contrast to NLOT, which is totally dependent on olfactory input, the remaining AMY is highly developed in anosmatic (odontocetes) and microsmatic (mysticetes and human) species. This implies that the AMY receives information inputs of different kind, such as auditory, in addition to olfactory stimuli. Compared to primates, dolphins appear to have an even larger AMY overall (Schwerdtfeger et al., 1984). The nuclear group with the largest dimensions was the basolateral nuclear group, which was made up of the paralaminar, lateral, basal, and accessory basal nuclei. In the examined dolphins, it was possible to identify the same parcellation of the amygdala present in other mammalian species (McDonald, 2003; Sah et al., 2003; Amunts et al., 2005; Morgan and Amaral, 2014), according to the nomenclature introduced by Price et al. (1987). Previous studies have shown that although the amygdaloid complex of different mammal species shares common characteristics, there is a clear difference in the organization, parcellation, and in the total and relative size of each amygdaloid body. Hence, this work represents the first classification of the AMY in the species striped dolphin, common dolphin, and Atlantic spotted dolphin, according to the currently accepted classification. Previous classification differs significantly from the one we have today. In addition, a discrepancy has been detected in the divisions commonly used in literature and the one proposed in the Nomina Anatomica Veterinaria (International Committee on Veterinary Gross Anatomical Nomenclature) and the International (human) Neuroanatomical Terminology (Table 3). Twelve nuclei have been found, arranged into three groups: the deep nuclei, the superficial area, and the remaining areas.

#### 4.1.1 The basolateral complex or deep nuclei

In the examined dolphins, this group displayed the largest size, as shown in other examined animals (McDonald, 2003). The lateral nucleus (NL) is the largest amygdaloid nucleus and receives the largest number of auditory and visual sensory information from the cortex and thalamus, and provides the most of the projections to the rest of the AMY (Pitkänen and Kemppainen, 2002). Its relative size with respect to the other amygdaloid nuclei increases in the following order: rat < monkey < man (Price et al., 1987). As observed in the porpoise and in the bottlenose dolphin (Breathnach and Goldby, 1954; Rambaldi et al., 2017), the NL was the largest, by volume and rostro-caudal extension. Its large volume is probably associated with the advanced auditory functions of these animals. Its presence was appreciated from the most caudal portion of the claustrum to the hippocampal formation. As described by Breathnach and Goldby (1954), the NL was evident for its neurons of homogenous distribution, medium to medium-large size, and intense affinity to thionine. Laterally, it was surrounded by the external capsule and medially by the NB and the NBA. Ventrally, the NL was delimited by the PL and the entorhinal cortex. The NL presented two divisions: principal and accessory. Interestingly, this nucleus has recently been characterized in the bottlenose dolphin and six main subdivisions have been identified as follows: lateral, ventromedial, dorsomedial, central, intermediomedial, and paracapsular (Rambaldi et al., 2017). Authors suggest that the presence of a higher number of subdivisions may indicate an exceptional capacity to elaborate and imbue external stimuli with emotional significance. In fact, sensory (somatosensory, acoustic, visual, and visceral) and mnemonic stimuli are received by the NL and integrated through interdivisional and intradivisional connections. The literature only reports two subdivisions in humans (Sorvari et al., 1998; Pitkanen and Kemppainen, 2002), four in monkeys (Pitkanen and Amaral, 1994), and three in rats (Pitkanen et al., 1995). The basal nucleus (NB) presented three divisions: parvicelular (small neurons), intermediate (medium size neurons), and magnocellular (large neurons). Morgane and Jacobs (1972) did not identify the intermediate division of the NB in the bottlenose dolphin (Morgane and Jacobs, 1972). The three subdivisions showed a high affinity to thionine staining. The NBmc was very evident by the size and intensity of coloration of its neurons, as well as by the total volume of this division, according to its description in the porpoise (Breathnach and Goldby, 1954). The NB was located lateral to the superficial nuclei and medially to the NL. The accessory basal nucleus (NBA) has been described in the porpoise (Breathnach and Goldby, 1954) and in bottlenose dolphin (Morgane and Jacobs, 1972; Rambaldi et al., 2017). However, according to Jansen (1953), this nucleus would not be present in the fin whale. The paralaminar nucleus (PL), present and well developed in human and non-human primates (deCampo and Fudge, 2012), in rat and cat (Price et al., 1987), and in the bat (Humphrey, 1936), was also observed in the examined dolphins.

#### 4.1.2 The cortical or superficial areas

These nuclei, of cortical origin, showed a stratified arrangement of their neurons. Previous studies in the porpoise, bottlenose dolphin and fin whale (Jansen, 1953; Breathnach and Goldby, 1954; Morgane and Jacobs, 1972) did not establish a differentiation between anterior cortical and posterior cortical nuclei, simply describing the presence of a “cortical nucleus,” dorsal to the magnocellular portion of the basal nucleus. The Nomina Anatomica also recognizes a single “*nucleus corticalis.*” However, our research allowed to uniquely identify each of the two nuclei in the dolphins under examination. Comparable to what has been documented in the literature for other species, including the bottlenose dolphin (Rambaldi et al., 2017), the anterior cortical nucleus (CoA) was located in the rostral portion of the amygdala, dorsally to the periamygdaloid cortex and medially to the basolateral complex. The posterior cortical nucleus (Cop) was located in the caudal portion of the AMY, dorsal to the AHA. The peri-amygdaloid area/cortex (PAC), was situated in the rostral half of the AMY, medially, in between the substantia innominata and the basolateral complex. According to the research done thus far, this nucleus has not been described in any cetacean species. The medial nucleus (M), in accordance with other cetacean species, was found limited medially by the substantia innominata and the optic tract and laterally by the NB (Jansen, 1953; Breathnach and Goldby, 1954; Morgane and Jacobs, 1972). It should be mentioned that the medial limit of the CeA was defined by the most dorsal and lateral part of the medial nucleus, as described by the same aforementioned authors.

#### 4.1.3 Remaining areas

The study also identified the following other remaining areas: the intercalated nuclei, the anterior amygdaloid area, the anterior amygdalo-hippocampal area, and the central nucleus, whose discussion deserves a separate section. Located dorsal to the basolateral complex in the most rostral part of the amygdala, the anterior amygdaloid area (AAA) was challenging to define. The location of the AAA has also been previously reported in porpoises (Breathnach and Goldby, 1954). It was identified by the presence of disorganized neurons with a variety of morphologies that resembled those found in the striatum. The elongated shape and parallel distribution of its neurons distinguished the anterior amygdalo-hippocampal area (AHA), which was limited to the caudal half of the amygdala and situated medial and parallel to the NB. It was distinguished by the presence of large neurons that stained strongly with thionine. The intercalated nuclei (I) or intercalated cell masses, were scattered clusters of small neurons, also described in the porpoise (Breathnach and Goldby, 1954) and in the fin whale (Jansen, 1953). Although they were not initially reported in the bottlenose dolphin (Morgane and Jacobs, 1972) they have only lately been described as small clusters of little neurons confined between the fiber bundles dividing the lateral and dorsomedial subdivisions of the NL (Rambaldi et al., 2017).

Neither the bed nucleus of the accessory olfactory tract (BAOT) nor the nucleus of the lateral olfactory tract (NLOT) have been identified in our work. Jansen (1953), however, reported finding NLOT in fin whales. Breathnach and Goldby (1954) observed neurons in the porpoise that were consistent with those of the NLOT, but they did not specify or rule out the possibility of a clearly defined nucleus. The lack of these nuclei could be brought on by the involution of the olfactory structures in toothed whales (Oelschläger, 2008). The hippocampal formation, which was restricted caudally to the AMY, has demonstrated significant involution in comparison to neighboring structures. Specifically, there was a clear reduction in the dentate gyrus, as observed in other cetacean species (Breathnach and Goldby, 1954; Patzke et al., 2015; Sacchini et al., 2022). The description of the claustrum in the bottlenose dolphin, suggests that it shares similarities with other species (Cozzi et al., 2014).

### 4.2 The central nucleus of the amygdala

The majority of research on the CeA focuses on its interactions with other brain areas (Price and Amaral, 1981; Fung et al., 2011). These studies have primarily focused on rats and non-human primates in addition to humans. The CeA receives sensory information from the basolateral complex and projects it to the brainstem and hypothalamus, where it mediates fear behavior with autonomic and behavioral response signals (Sah et al., 2003). Its function is not limited to expressing fear; it also plays a role in learning fear and creating memories (Wilensky et al., 2006). It was also noted in this work that the presence of CeA was consistently associated with the NBmc, in accordance with the description in the bottlenose dolphin (Morgane and Jacobs, 1972). There are some disparities, though, regarding the CeA’s divisions. In fact, different authors have given different descriptions to different CeA subdivisions within a single species. There have been reports of two (Davis and Whalen, 2001), three (Sah et al., 2003), and up to four (Pitkänen and Kemppainen, 2002) subdivisions in rats (*Rattus norvegicus*). The CeA of primates and humans, on the other hand, clearly shows two divisions (Pitkänen and Kemppainen, 2002). Two distinct subdivisions of the CeA were identified in our dolphins: one was lateral and dorsal to the NL, and the other medial and dorsal to the NBmc. The Atlantic spotted dolphin, however, was devoid of the medial subdivision (this finding will be later examined in more detail). Neither the bottlenose dolphin nor the porpoise have been reported to show these two subdivisions (Morgane and Jacobs, 1972). In terms of neuronal distribution and morphotypes, the two subdivisions were identical. Depending on the animal species, the medial and lateral parts of the CeA have different neuropeptides and projections to other amygdaloid nuclei and brain structures (Pitkanen and Kemppainen, 2002). Therefore, further investigation is needed to fully understand the roles played by each of the two CeA divisions in dolphins. We have acquired quantitative parameters of the CeA of an adult male common dolphin, which served as a rough estimate of its size. Nevertheless, measuring and comparing areas and perimeters across the various species under study was not the goal of our research. It is believed that within a species, CeA is the same size in both sexes. In fact, previous studies suggest that the medial nucleus (Cooke and Woolley, 2005) and the NLOT are the only amygdaloid nuclei that exhibit sexual dimorphism (Guillamon and Segovia, 1997). Additionally, the size of the human CeA is used to predict the development of various mental disorders. A study on males found that individuals with disorders affecting emotional processing (psychopathy) had an increased size of CeA ranging from 10% to 30% (Boccardi et al., 2011). The size and presence of the CeA have been determined by the presence and size of the NBmc. In particular, the maximum size of the NBmc has implied the maximum development of the CeA, especially its medial division. The adult male Atlantic spotted dolphin under examination was the only one whose AMY did not exhibit the medial portion of the CeA, making this data impractical. The CeA was smaller than that of the other species even after the NBmc reached its largest size. We have linked these data to the reported behavior of this species, which is less elusive and more gregarious when interacting with humans, than other species, such as the striped dolphin (Herzing, 1997). The authors have also firsthand experienced with this conduct during whale-watching boat excursions. This kind of behavior would be less common in adult males and more common in juveniles and mothers with their calf (Herzing, 1997). To support this theory and investigate the relationship between the size of the AMY and CeA, as well as the extent to which different toothed whales display gregarious behaviors and possible stress responses resulting from human interaction, more research is required. Since studies have shown that the AMY in humans decreases with age, another theory would be to relate the animal’s age to the size of the CeA (Bickart et al., 2011). The CeA is distinct from other amygdaloid nuclei because it originates from the striatum (McDonald, 1982; Schiess et al., 1999). This was also evident in the CeA neurons, strikingly similar to those of the FStr as previously reported in the bottlenose dolphin and porpoise (Breathnach and Goldby, 1954; Morgane and Jacobs, 1972). Large polygonal neurons were dispersed throughout the CeA or through the white matter space between the CeA and the NL. Similarities between LP neurons and those in the adjacent substantia innominata were observed. This is probably due to the origin of some CeA neurons from the substantia innominata (McDonald, 2003).

#### 4.2.1 Role of calbindin-D28k in the characterization of the CeA

The specific function of the protein calbindin-D28k, which binds calcium, is still unknown. It is found in a variety of central nervous system cell types and functions as helpful marker for different neuronal populations and subpopulations within different nuclei (McDonald, 1997). In fact, some authors have described the use of c-D28k to identify distinct amygdaloid nuclei as well as other brain areas such as the hippocampus, different cortical areas, or the cerebellum’s Purkinje cells (Seress et al., 1993; Ishikawa et al., 1995; McDonald, 1997; Mikkonen et al., 1997; Bombardi et al., 2006). Higher levels of c-D28k immunoreactivity are seen in the CeA and superficial nuclei compared to the deep nuclei, and the CeA also exhibits strong neuropil labeling (Sorvari et al., 1996). The CeA was successfully identified by using c-D28k immunohistochemistry; however, different labeling patterns were observed in some of the animals. As such, c-D28k enabled the precise definition of the CeA’s boundaries. On the other hand, a variable distribution of c-D28k immunoreactivity in the CeA was noted in certain instances. Variations in c-D28k staining may be caused by postmortem time and tissue protein deterioration. However, rats, primates, and humans have all been shown to exhibit a heterogeneous distribution of c-D28k positive neurons inside the CeA, nevertheless (McDonald, 1997; Pitkanen and Kemppainen, 2002). In summary, previous work (Pitkanen and Kemppainen, 2002) has shown that in order to comprehend the marking patterns found in the various amygdaloid nuclei and their subdivisions, it is imperative to compare and contrast cyto-architectonic and chemo-architectonic observations in various animal species. Further investigation is necessary to characterize the distribution of positive neurons along the rostro-caudal extension of the CeA, as well as in its lateral and medial subdivisions.

### 4.3 Limitation of the study

Cetaceans in general, and dolphins in particular, are wild marine animals and neuroanatomical studies in these mammals are often very challenging. The postmortem period degrades the quality of the tissues, and stereological studies are difficult to carry out because some of the samples from various brain regions need to be designated for neuropathological examinations. In addition, fixation by immersion has been often deemed inadequate, particularly in large brains where the fixative is slow to penetrate. Inadequate or late fixation of subcortical structures led to low tissue quality, sections more prone to rupturing, poor c-D28k immunoreactivity and a very low affinity for thionine. Actually, because of the poor quality of their AMY, we had to exclude four toothed whales, namely two bottlenose dolphin, one short-finned pilot whale (*Globicephala macrorhynchus*, Gray 1846, family Delphinidae), and one Blainville’s beaked whale (*M. densirostris*, De Blainville 1817, family Ziphiidae) (Sacchini, 2015). In the study of the AMY, one of the main difficulties has been its deep location inside the brain, compared to cortical areas. Its position represents a limit for the penetration of the fixative. In some cases, immunoreactivity against c-D28k has not been homogeneous, with only small areas of positivity being detected. The AMY which provided the most comprehensive description were those with the best tissue quality, which resulted in improved thionine staining, and a strong and uniform immunoreaction to c-D28k. Further complicating the investigation is the small size of CeA’s neurons and their poor affinity to thionine. The morphometric study was then carried out on the animals with the best thionine staining: the common dolphin and the two striped dolphins. Furthermore, CeA areas and perimeters were reported only in one common dolphin. Any estimation of the CeA’s size was only approximative because its limits were frequently not well defined, according to other authors (Breathnach and Goldby, 1954). Future studies should employ additional calcium binding proteins and a larger range and number of cetacean species in order to deepen our understanding of the AMY in these marine mammals.

## Data availability statement

The original contributions presented in this study are included in this article/supplementary material, further inquiries can be directed to the corresponding author.

## Ethics statement

This investigation relies solely on post-mortem analysis, so ethical review and approval were not required. The Spanish Ministry of Environment and the Canary Islands Government’s environmental department granted the necessary authorization for the management of stranded cetaceans. Live animals were not used in any of the experiments.

## Author contributions

SS: Conceptualization, Investigation, Methodology, Resources, Validation, Visualization, Writing – original draft, Writing – review & editing. CB: Conceptualization, Investigation, Methodology, Supervision, Validation, Writing – review & editing. MA: Data curation, Resources, Supervision, Writing – review & editing. PH: Conceptualization, Funding acquisition, Investigation, Project administration, Supervision, Writing – review & editing.

## Footnotes

1
www.ensembl.org
